# Association of repeatedly measured intermediate risk factors for complex diseases with high dimensional SNP data

**DOI:** 10.1186/1748-7188-5-17

**Published:** 2010-02-11

**Authors:** Sandra Waaijenborg, Aeilko H Zwinderman

**Affiliations:** 1 Department of Clinical Epidemiology, Biostatistics and Bioinformatics, Academic Medical Center, University of Amsterdam, Meibergdreef 9, 1100 DD Amsterdam, the Netherlands

## Abstract

**Background:**

The causes of complex diseases are difficult to grasp since many different factors play a role in their onset. To find a common genetic background, many of the existing studies divide their population into controls and cases; a classification that is likely to cause heterogeneity within the two groups. Rather than dividing the study population into cases and controls, it is better to identify the phenotype of a complex disease by a set of intermediate risk factors. But these risk factors often vary over time and are therefore repeatedly measured.

**Results:**

We introduce a method to associate multiple repeatedly measured intermediate risk factors with a high dimensional set of single nucleotide polymorphisms (SNPs). Via a two-step approach, we summarized the time courses of each individual and, secondly apply these to penalized nonlinear canonical correlation analysis to obtain sparse results.

**Conclusions:**

Application of this method to two datasets which study the genetic background of cardiovascular diseases, show that compared to progression over time, mainly the constant levels in time are associated with sets of SNPs.

## Background

Among the examples of complex diseases, several of the major (lethal) diseases in the western world can be found, including cancer, cardiovascular diseases and diabetes. Increasing our understanding of the underlying genetic background is an important step that can contribute in the development of early detection and treatment of such diseases. While many of the existing studies have divided their study population into controls and cases, this classification is likely to cause heterogeneity within the two groups. This heterogeneity is caused by the complexity of gene regulation, as well as many extra- and intracellular factors; the same disease can be caused by (a combination of) different pathogenetic pathways, this is referred to as phenogenetic equivalence. Due to this heterogeneity, the genetic markers responsible for, or involved in the onset and progression of the disease are difficult to identify [1]. Moreover, the risk of misclassification is increased if the time of onset of the disease varies.

In order to overcome these problems, rather than dividing the study population into cases and controls, it is preferable to identify the phenotype of a complex disease by a set of intermediate risk factors. Because of the high diversity of pathogenetic causes that can lead to a complex disease, such intermediate risk factors are likely to have a much stronger relationship with the measured genetic markers. Intermediate risk factors can come in a number of varieties, as broad as the whole gene expression pattern of an individual up to as specific as a set of phenotypic biomarkers chosen based upon prior knowledge of the diseases, e.g., lipid profiles as possible risk factors for cardiovascular diseases. These risk factors often vary over time and are therefore repeatedly measured.

In recent studies we have used penalized canonical correlation analysis (PCCA) to find associations between two sets of variables, one containing phenotypic and the other containing genomic data [2,3]. PCCA penalizes the two datasets such that it finds a linear combination of a selection of variables in one set that maximally correlates with a linear combination of a selection of variables in the other set; thereby making the results more interpretable. Highly correlated variables, caused by eg. co-expressed genes, are grouped into the same results.

Although canonical correlation analysis accounts for the correlation between variables within the same variable set, CCA is not capable of taking advantage of the simple covariance structure of the longitudinal data. Our goal was to provide biological and medical researchers with a much needed tool to investigate the progression of complex diseases in relationship to the genetic profiles of the patients. To achieve this, we introduce a two-step approach: first we summarize each time course of each individual and, secondly, we apply penalized canonical correlation analysis, where the uncertainty of the summary estimates is taken into account by using weighted-least squares. Additionally, optimal scaling is applied such that qualitative variables can be used within the PCCA, resulting in penalized nonlinear CCA (PNCCA) [3]; e.g., for transforming single nucleotide polymorphisms (SNPs) into continuous variables such that they capture the measurement characteristics of the SNPs. By adapting these approaches, we are able to extract groups of categorical genetic markers that have a high association with multiple repeatedly measured intermediate risk factors.

To illustrate PNCCA, this method was applied to two datasets. The first dataset is part of the Framingham Heart Study http://www.framinghamheartstudy.org, which contains information about repeatedly measured common characteristics that contribute to cardiovascular diseases (CVD), together with genetic data of about 50,000 SNPs. These data were provided for participants to the genetic analysis workshop 16 (GAW16). The second dataset is the REGRESS dataset [4], which contains information about lipid profiles together with about 100 SNPs located in candidate genes. By applying PCCA, we were able to extract groups of SNPs which were highly associated with a set of repeatedly measured intermediate risk factors. Cross-validation was used to determine the optimal number of SNPs within the selected SNP clusters.

## Results and Discussion

### Framingham heart study

The Framingham heart study was performed to study common characteristics that contribute to cardiovascular diseases (CVD). Besides information about these risk factors, the study contains information about genetic data of about 50,000 single nucleotide polymorphisms (SNPs). Risk factors were measured from the start of the study in 1948 up to four times, every 7 to 12 years. Three generations were followed, however, to have consistent measurements, only the individuals of the second generation were included in this study. The data of the Framingham heart study were provided for participants to the genetic analysis workshop 16 (GAW grant, R01 GM031575).

We considered the measurements of LDL cholesterol (mg/dl), HDL cholesterol (mg/dl), triglycerides (mg/dl), blood glucose (mg/dl), systolic and diastolic blood pressure and body mass index; each measured up to 4 times (in fasting blood samples). LDL cholesterol was estimated using the Friedewald formula: *LDL cholesterol *= *total cholesterol - HDL cholesterol *- 0.2*triglycerides. Furthermore, we considered the data of the affymetrix 50 K chip containing about 50,000 SNPs.

The offspring generation consists of 2,583 individuals over the age of 17, of which 157 suffered from a coronary heart disease (of which 2 before the beginning of the study). From this data 3 individuals had a negative LDL cholesterol level and were therefore removed from the data, together with 27 individuals who had less than 2 observations for one or more of the 7 intermediate risk factors. 7 individuals were removed because they were missing more than 5% of their genetic data. Monomorphic SNPs and SNPs with a missing percentage of 5% or more were deleted from further analysis, remaining missing data were randomly imputed based only on the marginal distribution of the SNP in all other individuals. Because our primary interest concerned common SNP variants, we therefore grouped SNP classes with less than 1% observations, with its neighboring SNP class; i.e., we grouped homozygotes of the rare allele together with the heterozygotes. This resulted in a dataset consisting of 2,546 individuals, 7 intermediate risk factors and 37,931 SNPs.

Penalized nonlinear canonical correlation analysis was used to identify SNPs that are associated with a combination of intermediate risk factors of cardiovascular diseases. Here for, the data was divided based upon subjects into two sets; one test set containing 546 subjects and an estimation set of 2,000 subjects to estimate the weights in the canonical variates, the transformation functions and to determine the optimal number of variables within the SNP dataset.

To remove the dependency within the longitudinal data, seven models were fitted, one for each of the seven intermediate risk factors. The individual change pattern in time of each of the seven intermediate risk factors was summarized with the best linear unbiased predictions (BLUP) of the intercept and slope parameters, using the following mixed effect model:

*y*_*it *_represents one of the seven risk factors of individual *i *measured at age *t*, *trt*_*it *_the treatment individual *i *received at age *t *and *sex*_*i *_the gender of the individual *i*. In the models for LDL cholesterol, HDL cholesterol, triglycerides, blood glucose and BMI, the treatment with cholesterol lowering medication was used as a covariate. In the models for systolic and diastolic blood pressure, blood pressure lowering medication was used. Here, *trt *= 0 stands for no medication and *trt *= 1 for pharmacological treatment. The measurements for both age as well as the risk factors were standardized to have mean zero.

A new dataset was formed, containing the random intercepts and the random slopes from each individual, for each of the seven intermediate risk factors. The random slopes and random intercepts of the blood glucose variable had a perfect correlation, indicating no time effect to be present. Therefore the slope variable of the blood glucose variable was removed from the newly obtained dataset, which resulted in a set containing 13 measures (7 random intercepts (*b*_0*i*_'s) and 6 random slopes (*b*_1*i*_'s) (see table 1)) and a weight set with 13 accompanying standard errors.

**Table 1:** Intermediate risk factors of the Framingham heart study.

	First canonical variate		Second canonical variate	
Phenotype	Loadings	Cross-loadings	Loadings	Cross-loadings
HDL intercept	0.76	0.18	-0.25	-0.10
HDL slope	-0.05	-0.02	0.07	0.03
LDL intercept	-0.16	-0.04	-0.12	-0.05
LDL slope	-0.07	-0.02	-0.08	-0.03
triglyceride intercept	-0.10	-0.03	-0.02	-0.01
triglyceride slope	0.15	0.04	0.11	0.04
blood glucose	0.02	0.01	0.65	0.25
systolic intercept	-0.07	-0.01	0.08	0.02
systolic slope	-0.11	-0.02	0.08	0.02
diastolic intercept	0.06	0.02	0.13	0.05
diastolic slope	-0.11	-0.03	0.16	0.06
BMI intercept	-0.05	0.00	0.73	0.30
BMI slope	0.07	0.02	0.71	0.28

The loadings and cross-loadings of the intermediate risk factors within the first and second canonical variate pair.

**Figure 1:**
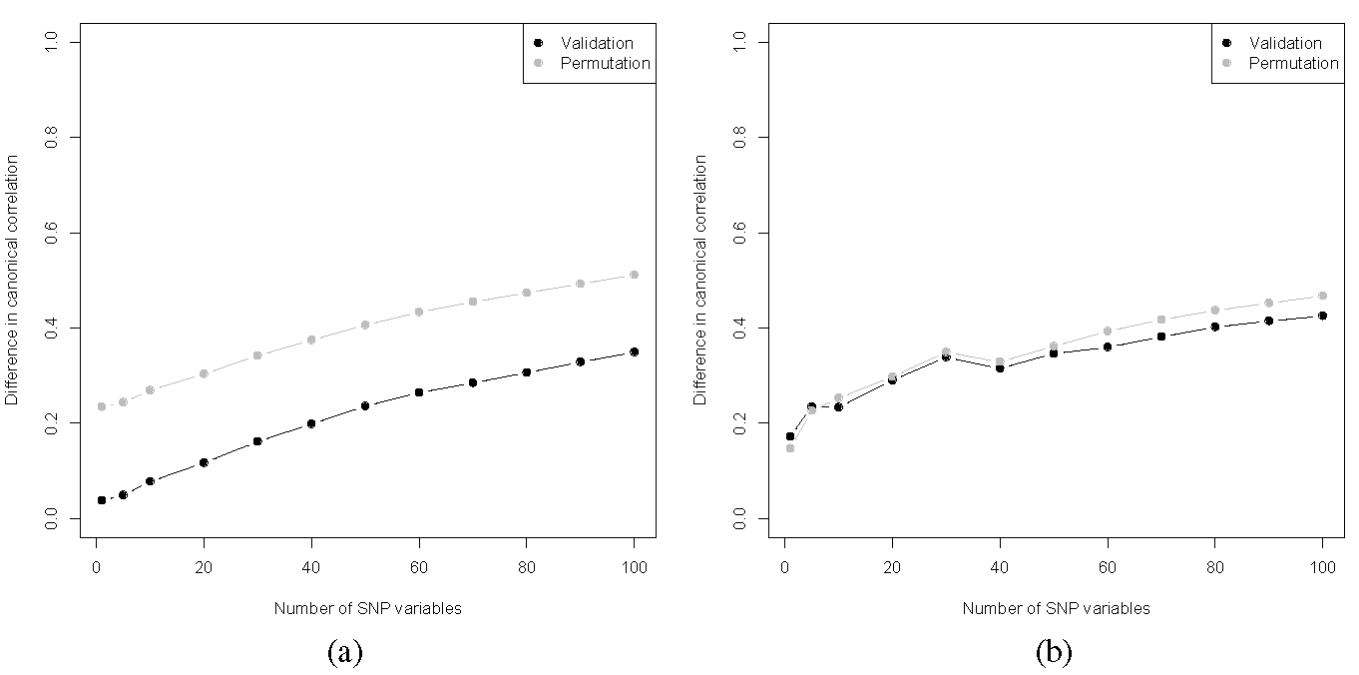
**Framingham heart study**. Optimization of the first (a) and second (b) canonical variate, for differing number of SNP variables.

**Figure 2:**
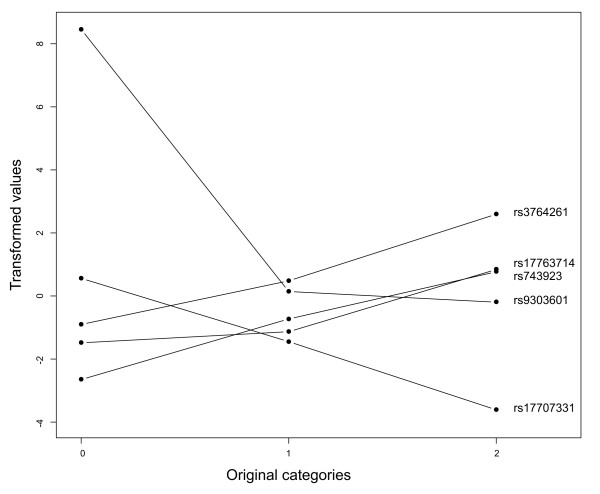
**Transformation of the selected SNPs**. Transformation of the selected SNPs in the Framingham heart study.

By means of 10-fold cross-validation, the optimal number of SNP variables was determined for several canonical variates (see figure 1). As can be seen in figure 1, with increasing number of selected variables, the difference between the canonical correlation of the validation and the training set also increased. For the first canonical variate pair (figure 1a), the difference between the canonical correlation of the permuted validation set and the training set was high, indicating that there were associating SNPs present in the dataset. Adding more variables to the model did not decrease the difference between validation and training sets, therefore, the number of important variables was very small. A model with 1 SNP variables was optimal, however, to be sure not to miss any important SNPs, we built a model containing 5 SNPs. PNCCA was next performed on the whole estimation dataset, obtaining 5 SNP variables associated with all the phenotypical intermediate risk factors, this resulted in a model with a canonical correlation of 0.24. The weights and transformations of this optimal model were applied to the test set, resulting in a canonical correlation of 0.17. The loadings (correlations of variables and their respective canonical variates) and cross-loadings (correlations of variables with their opposite canonical variate) are given in tables 1 and 2 for the intermediate risk factors and selected SNPs, respectively. In figure 2 the transformations of the selected SNP variables are given, it can be seen that almost all SNPs had an additive effect, except for SNP *rs9303601*, which had a recessive effect.

**Table 2:** Selected SNPs in the Framingham heart study.

Chrom	Position	ID	Gene symbol	Loadings	Cross-loadings
First canonical variate					

6	36068368	rs17707331	*SLC26A8*	0.03	0.10
6	36088099	rs743923	*SLC26A8*	0.02	0.10
8	62213297	rs17763714		0.02	0.10
16	55550825	rs3764261	near *CETP*	1.00	0.23
17	39634367	rs9303601		-0.00	0.10

Second canonical variate					

1	54388154	rs11576359	*CDCP2*	0.07	0.09
1	70656428	rs1145920	*CTH*	0.17	0.10
1	82015671	rs12072054		0.09	0.09
2	28371365	rs4666051	*BRE*	0.15	0.10
2	33358643	rs2290427	*LTBP1*	0.11	0.09
2	49052952	rs12713027	*FSHR*	0.41	0.10
2	49074650	rs4494802	*FSHR*	0.67	0.14
2	177267412	rs16864244	*LOC375295*	0.08	0.09
3	122662360	rs669277	*POLQ*	0.15	0.09
3	122669112	rs532411	*POLQ*	0.14	0.09
4	178830262	rs13149928		0.19	0.10
5	79069147	rs2278240	*CMYA5*	0.19	0.10
6	33380833	rs2071888	*TAPBP*	0.13	0.10
6	42374980	rs4714595	*TRERF1*	0.06	0.08
6	102197213	rs6925691	*GRIK2*	0.25	0.11
6	116504475	rs12527159		0.23	0.10
7	116990653	rs213952	*CFTR*	0.11	0.10
7	129737976	rs2171492	*CPA4*	0.22	0.10
7	129771213	rs7786598	*CPA5*	0.27	0.09
7	129772024	rs1532047	*CPA5*	0.29	0.10
7	137639294	rs410156		0.08	0.09
8	23574003	rs7006278		0.07	0.09
10	71959784	rs2275060	*KIAA1274*	0.15	0.09
10	81916682	rs1049550	*ANXA11*	0.18	0.10
11	12036827	rs2403569		0.07	0.08
11	24693192	rs2631439	*LUZP2*	0.14	0.09
11	33438534	rs2615913		0.20	0.10
11	92329680	rs7936247		0.11	0.09
11	101990763	rs7126560	*MMP20*	0.23	0.10
11	133808516	rs7949167		0.07	0.08
12	7040597	rs12146727	*C1S*	0.23	0.10
12	14873619	rs3088190	*ART4*	0.11	0.09
13	95455188	rs16951415	*UGCGL2*	0.13	0.09
14	59141031	rs10483717	*RTN1*	0.29	0.11
15	64238853	rs4776752	*MEGF11*	0.15	0.09
16	15548316	rs9930648	*C16orf45*	0.05	0.08
16	77671528	rs12935535	*WWOX*	0.11	0.09
17	53939507	rs2302190	*MTMR4*	0.14	0.10
19	38606005	rs11084731	*PEPD*	0.29	0.11
22	37171988	rs196084	*KCNJ4*	0.09	0.09

Selected SNPs within the first and second canonical variate pair, together with their loadings and cross-loadings.

The first canonical variate pair showed a strong association between the HDL intercept and SNP *rs3764261*, which is closely located to the *CETP *gene and has been reported to be associated with HDL concentrations [5]. The low loadings of the other SNPs show their small contribution to the first canonical variate of the SNP, this confirmed our results of the optimization step, which indicated that one SNP would be sufficient. Based on the loadings and cross-loadings, the canonical variate of the intermediate risk factors also seems to be constructed of one variable only, namely the HDL intercept.

Based upon the residual estimation matrix, the second canonical variate pair was obtained in a similar fashion via cross-validation. For small numbers of variables the predictive performance was limited (see figure 1b), which was represented by the overlap between the results of the validation and the permutation sets. With larger number of SNPs (>40) a clearer separation between the validation and the permutation set appeared, but the difference in canonical correlation also increased. We therefore chose to make a model with 40 SNPs.

Penalized CCA was next performed on the whole (residual) estimation set to obtain a model with 40 SNP variables associated with all the intermediate risk factors, this resulted in a model with a canonical correlation of 0.40, and a canonical correlation in the (residual) test set of 0.02. This shows the importance of the permutation tests; as we could already see by the overlap between the validation and the permutation results in figure 1b, the predictive performance of the model was expected to be poor as was confirmed by the canonical correlation of the test set.

Although the loadings and cross-loadings for some of the SNPs (*rs12713027 *and *rs4494802*, both located in the follicle stimulating hormone receptor) and intermediate risk factors (blood glucose and BMI) were quite high, no references could be found to confirm these associations.

Because the second canonical variate pair was hardly distinguishable from the permutation results, we did not obtain further variate pairs.

### REGRESS data

The Regression Growth Evaluation Statin Study (REGRESS) [4] was performed to study the effect of 3-hydroxy-3-methylglutaryl coenzyme A reductase inhibitor pravastatin on the progression and regression of coronary atherosclerosis. 885 male patients, with a serum cholesterol level between 4 and 8 mmol/l, were randomized to either treatment or placebo group. Levels for HDL cholesterol, LDL cholesterol and triglycerides were measured repeatedly over time, at baseline (before treatment) and 2, 4, 6, 12, 18 and 24 months after the beginning of the treatment. For each patient 144 SNPs in candidate genes were determined, after removing monomorphic SNPs and SNPs with more than 20% missing data, 99 SNPs remained and missing data were imputed. Individuals without a baseline measurement and individuals with less than 2 follow-up measurements and/or more than 10% missing SNPs were excluded from the analysis. The final dataset contained 675 individuals together with 99 SNPs located in candidate genes and 3 intermediate risk factors.

The dataset was divided into two sets, one estimation set with 500 subjects and a test set of 175 subjects. To remove the dependency within the longitudinal data, each of the three intermediate risk factors was summarized into two summary measures, a random intercept and a random slope, using the following mixed effect model:

*y*_*i*0 _was the measurement of risk factor *y *taken at baseline for patient *i*; i.e. the time point before medication was given. *trt *was either placebo or pravastatin. The measurements for both age as well as the risk factor at time point zero and the risk factors were standardized to have mean zero. The random slopes and random intercepts of LDL cholesterol, HDL cholesterol and triglyceride formed set **Y**.

**Figure 3:**
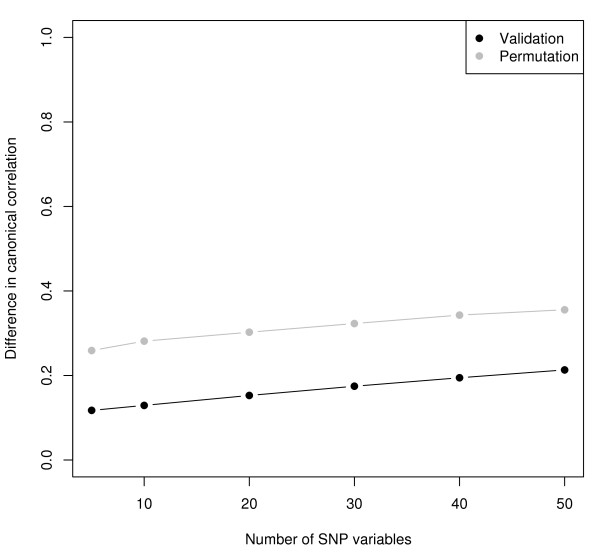
**REGRESS study**. Optimization of the first canonical variate, for differing number of SNP variables.

Via 10-fold cross-validation the optimal number of SNP variables was determined (see figure 3). As can be seen from figure 3, the optimal number of variables was 5. The model containing 5 SNPs had a canonical correlation of 0.23 in the whole estimation set and a canonical correlation of -0.04 in the test set. The loadings and cross-loadings are given in tables 3 and 4, for the selected SNPs and risk factors, respectively. All the selected SNPs are located in the *CETP *gene, the obtained canonical variate correlated mostly with the HDL intercept. These results are quite similar to the results of the Framingham heart study, where a SNP closely located to the *CETP *gene highly associated with the HDL intercept.

**Table 3:** Selected SNPs in the REGRESS study.

Gene symbol	ID	Polymorphism	Loadings	Cross-loadings
*CETP*	rs12149545	G-2708	0.79	0.17
*CETP*	rs708272	TaqIB	0.96	0.20
*CETP*		CCC+784A	0.89	0.19
*CETP*		Msp I	0.47	0.17
*CETP*	rs1800775	C-629A	0.94	0.22

Selected SNPs within the first canonical variate pair, together with their loadings and cross-loadings.

**Table 4:** Intermediate risk factors of the REGRESS study.

Phenotype	Loadings	Cross-loadings
HDL intercept	0.75	0.17
HDL slope	-0.14	-0.03
LDL intercept	-0.19	-0.03
LDL slope	-0.11	-0.03
triglyceride intercept	0.27	0.07
triglyceride slope	0.28	0.07

The loadings and cross-loadings of the intermediate risk factors within the first canonical variate pair.

The residual matrix for the intermediate risk factors was determined and while obtaining the second canonical variate, the SNPs selected in the first canonical variate were fixed at their optimal transformation. The validation and permutation results were overlapping (data not shown), so no further information could be obtained from this dataset.

## Conclusions

We have introduced a new method to associate multiple repeatedly measured intermediate risk factors with high dimensional SNP data. In this paper we have chosen to summarize the longitudinal measures into random intercept and random slopes via mixed-effects models. Mixed-effects models deal with intra-subject correlation by allowing random effects in the models, these models focus on both population-average and individual profiles by taking the dependency between repeated measures into account. Due to the high number of possible models, they can be too restrictive in the assumed change over time. Further, these models need many assumptions for the underlying model.

Other techniques to summarize longitudinal profiles, like area under the curve, average progress, etc., focus mainly on certain aspects of the response profile, or fail in the presence of unbalanced data. Often they lose information about the variability of the observations within patients. The pros and cons of summary statistics should be weighed to come up with the best solution, our decision to use mixed-effect models was based on the fact that the data showed a linear trend and because there was unbalanced data; i.e., unequal number of measurements for the individuals and the Framingham heart study measurements were not taken at fixed time points.

To make the results more interpretable, we chose only to penalize the **X**-side containing the SNPs. The number of intermediate risk factors was sufficiently small such that penalizing the number of variables would not increase the interpretation. While modeling the second canonical variate pair, a small ridge penalty was added to the **Y**-side to overcome the multicollinearity caused by the removal of the information of the first canonical variate.

Alternative methods for our two-step approach include performing penalized CCA without considering the fact that variables are repeatedly measured. This can be reasonable in the case of clinical studies, where one wants to see if changes at a certain time point after the beginning of a treatment are associated with certain risk factors. However, in observational studies fixed time points are difficult to obtain and getting a matrix without too much missing data is almost impossible, due to the diversity of time points at which a measurement can be obtained. Another option might be to summarize each repeatedly measured variable and associate them separately with the SNP data via a regression model in combination with the elastic net and optimal scaling. However, this method does not take the dependency between the intermediate risk factors into account and moreover, it can transform each SNP variable differently; which makes it difficult to integrate the results of the different regression models.

The residual matrix of the **X**-side, achieved by fixing the transformed variables in their primary transformed optimal form, was optional. In studies with small numbers of SNP variables, like in the case of the REGRESS study, fixation is preferred to overcome the same variable to be optimized twice. For studies like the Framingham heart study, fixation is not necessary, since there is almost no overlap between the selected SNPs in succeeding canonical variate pairs.

Strikingly, both studies showed an association between SNPs located near or in the *CETP *gene and the HDL intercept. Neither of the datasets could find other associations, which could be explained by the absence of important (environmental) factors, or by the fact that SNP effect is more complicated and more complex models are necessary to model this effect. The results in both studies show that the random intercepts get the highest loadings and cross-loading, while the random slopes seem to be less associated with the selected SNPs. This could indicate that individuals average values are to some extent genetically determined, while the changes over time are influenced by other factors, e.g. environmental factors.

The selected SNPs within the first canonical variate pairs are consistent with results found in literature [6], however, the reproducibility is quite low, especially in the REGRESS study where canonical correlation of the test set came close to zero. It seems that the bias caused by univariate soft-thresholding has considerable impact on the weight estimation and therefore predictive performance is quite low, especially in studies where the canonical correlation is already low due to the absence of important variables. Our method is especially useful as a primarily tool for gene discovery, such that biologists have a much smaller subset for deeper exploration, and not so much as to make predictive models.

## Methods

Our focus lies on intermediate risk factors, we assume that individuals with similar progression-profiles of the intermediate risk factors share the same genetic basis. By associating a dataset with repeatedly measured risk factors and a dataset with genetic markers, we can extract the common features out of the two sets. Canonical correlation analysis can be used to extract this information. However, the fact that one dataset contains categorical data and the other contains multiple longitudinal data complicates the data analysis. In the next section we give a summary of the penalized nonlinear canonical correlation analysis (PNCCA), more details about this method can be found in [2] and [3]. Hereupon, we extend the PNCCA such that it can handle longitudinal data. Finally, the algorithm will be presented.

### Canonical correlation analysis

Consider the *n *× *p *matrix **Y **containing *p *intermediate risk factors, and the *n *× *q *matrix **X **containing *q *SNP variables, obtained from *n *subjects. Canonical correlation analysis (CCA) captures the common features in the different sets, by finding a linear combination of all the variables in one set which correlates maximally with a linear combination of all the variables in the other set. These linear combinations are the so-called canonical variates *ω *and *ξ*, such that *ω *= **Yu **and *ξ *= **Xv**, with the weight vectors **u***' *= (*u*_1_, ..., *u*_*p*_) and **v***' *= (*v*_1_, ..., *v*_*q*_). The optimal weight vectors are obtained by maximizing the correlation between the canonical variate pairs, also known as the canonical correlation.

When dealing with high-dimensional data, ordinary CCA has two major limitations. First, there will be no unique solution if the number of variables exceeds the number of subject. Second, the covariance matrices **X**^*T*^**X **and **Y**^*T*^**Y **are ill-conditioned in the presence of multicollinearity. Adapting standard penalization methods, like ridge regression [7], the lasso [8], or the elastic net [9], to the CCA could solve these problems. Via the two-block Mode B of Wold's original partial least squares algorithm [10,11], the CCA can be converted into a regression framework, such that adaptation of penalization methods becomes easier. Wold's algorithm performs two-sided regression (one for each set of variables), therefore either of the two regression models can be replaced by another optimization method, such as one-sided penalization or different penalization methods for either set of variables.

#### Penalized canonical correlation analysis

In genomic studies the number of variables often greatly exceeds the number of subjects, causing overfitting of the models. Moreover, due to the high number of variables interpretation of the results is often difficult. Previously, we and others [2,12,13] have shown that adapting univariate soft-thresholding [9] to CCA makes the interpretation of the results easier by extracting only relevant variables out of high dimensional datasets. Univariate soft-thresholding (UST) provides variable selection by imposing a penalty on the size of the weights. Because UST disregards the dependency between variables within the same set, a grouping effect will be obtained. So groups of highly correlated variables will be selected or deleted as a whole. UST can be applied to one side of the CCA-algorithm for instance the SNP dataset; the weights **v **belonging to the *q *SNP variables in matrix **X **are estimated as follows:

with *f*_+ _= *f *if *f *> 0 and *f*_+ _= 0 if *f *≤ 0, and *λ *the penalization penalty.

#### Penalized nonlinear canonical correlation analysis

When dealing with categorical variables (like SNP data), linear regression does not take the measurement characteristics of the categorical data into account. We previously developed penalized nonlinear CCA (PNCCA) [3] to associate a large set of gene expression variables with a large set of SNP variables. The set of SNP variables was transformed using optimal scaling [14,15]; each SNP variable was transformed into one continuous variable which depicted the measurement characteristics of that SNP, and subsequently this was combined with UST.

Each SNP has three possible genotypes; (a) wildtype (the common allele), (b) heterozygous and (c) homozygous (the less common allele). The measurement characteristics of these genotypes were restricted to have an additive, dominant, recessive or constant effect; this knowledge determined the ordering of the corresponding transformed variables. Each SNP variable can have one of the following restriction orderings:

• Additive effect:  or

• Recessive effect:  or

• Dominant effect:  or

• Constant effect: ,

with ℑ_*j *_the transformation function of SNP *j*, *x*_*a*_: wildtype, *x*_*b*_: heterozygous and *x*_*c*_: homozygous and  the transformed value for category *a *for variable *j*. The effect of the heterozygous form of SNP *j *always lies between the effect of the wildtype and homozygous genotype.

Optimal transformations of the SNP data can be achieved through the CATREG algorithm [14]. Let **G**_*j *_be the *n *× *g*_*j *_indicator matrix for variable *j *(*j *∈ (1, ... *q*)), with *g*_*j *_the number of categories of variable *j*. And let **c**_*j *_be the categorical quantifications of variable *j*. Then the CATREG algorithm with univariate soft-thresholding will look as follows:

**For each variable ***j*, *j *= 1, ..., *q*

(1) Obtain unrestricted transformation of **c**_*j*_

(2) Restrict (according to the restriction orderings given above) and normalize  to obtain 

(3) obtain the transformed variable 

(4) Perform univariate soft-thresholding (UST)

### Longitudinal data

Although CCA accounts for the correlation between variables within the same set, it neglects the longitudinal nature of the variables. CCA uses a general covariance structure and cannot directly take advantage of the simple covariance structure in longitudinal data. Furthermore, it does not deal well with unbalanced data, caused by e.g. measurements taken at random time points and drop-outs.

To remove the dependency within the repeated measures of each intermediate risk factor, we consider summary statistics that best capture the information contained in the repeated measures. Summary measures are used for their simplicity, since usually no underlying model assumptions have to be made and the summary measures can be analyzed using standard statistical methods. A large number of the summary measures focus only on one aspect of the response over time, but this can mean loss of information. Information loss should be minimized and depending on the question of interest, the summary measure should capture the most important aspects of the data. If all measurements are taken at fixed time points, summary measures like principal components of the different intermediate risk factors can be used. When additionally a linear trend can be seen in the data, simple summary statistics can be sufficient, like area under the curve, average progress, etc.

If variables are measured at random time points and/or have an unequal number of measurements and follow a linear trend, it can be best summarized into a linear model, by mixed-effects models [16]. The obtained random effects for intercept and slope, tells us how much each individual differs from the population average. Mixed-effects models account for the within-subject correlation, caused by the dependency between the repeated measurements. Let *y*_*it *_be the response of subject *i *at time *t*, with *i *= 1, ..., *N *and *t *= 1, ..., *T*_*i*_. For each risk factor the following model can be fitted:

with *b*_*i *_~ *N*(0, *D*) and *ε *~ *N*(0, *σ*_*i*_), *b*_*i *_and *ε *independent. The *β*_*j*_'s are the population average regression coefficients, which contains the fixed effects. **b**_*i *_are the subject specific regression coefficients, containing the random effects. The random effects *b*_*i*_'s tell use how much the individual's intercept (*b*_0*i*_) and slope (*b*_1*i*_) differ from the population's average. We assume that individuals with similar deviations from the population average have the same underlying genetic background. Therefore the random effects are used as a replacement of the repeated intermediate risk factors in the canonical correlation analysis on the **Y**-side. Consequently, **Y **no longer exists of an unbalanced set of variables, but is replaced by of a complete set of intercepts and slopes.

When additional information is available, like medication and sex, these can be added to the model:

where **Z **contains the covariates and *l *is the number of covariates.

Since the random effect are estimates and important information can be lost, the reliability of the random effects should be taken into account (see next section). Depending on the number of measurements and the complexity of the time trends, more complex change patterns can also be explained by the mixed-effects models.

### Weighted least squares

The longitudinal variables are summarized into a smaller number of variables, where each summary variable represents a certain property of the risk factor's trend. However, when summarizing the longitudinal variables, the reliability of the obtained summary variables varies between patients. The summary measures for individuals with no missing values and who were followed over a long time period, are more reliable than the summary measures of individuals who were followed for a shorter time period and/or have missing values (due to drop-out or intermediate missingness). This uncertainty is depicted in the standard errors of the summary statistics, in the case of mixed-effects models the standard errors of the random effects.

To make sure that summary measures with smaller standard errors contribute more to the estimation of the canonical weights; we use a weighted least squares regression, on the **Y**-side (the intermediate risk factor side) of the CCA algorithm. In some individuals certain intermediate risk factors can be measured more often than others, e.g., an individual can have four repeatedly measured LDL cholesterol values and only two blood glucose values. Therefore summary variables within an individual can get different uncertainties, and ordinary weighted least squares is no longer sufficient. To overcome this problem a backfitting procedure is used in which in each step of the iterative process an univariate weighted least squares regression model is fitted to estimate the canonical weights. This downweights the squared residuals for observations with large standard errors.

Suppose **W **is an *n *× *p *matrix, containing the reciprocals of the squared standard errors of the *p *summary variables. The estimation of the canonical weights **u **of the **Y**-side (summarized repeated measures) is done as follows:

1. Standardize **Y **and set starting values **u **= (1, 1, ..., 1)'.

2. Estimate **u **as follows

**Repeat loop across variables ***j*, *j *= 1, ..., *p*

(a) Remove the contribution of all variables except variable *j*

**z**_-*j *_= *ξ *- **u**_-*j*_**Y**_-*j*_

(b) Obtain the estimate of 

(c) Update **u**, with 

**until u **has converged.

In our analysis, matrix **W **contains the reciprocal of the squared standard errors of the random effects. Other weights can also be used, e.g. the number of times a risk factor is measured.

### Final algorithm

Our CCA method is able to deal with a large set of categorical variables (SNPs) and a smaller set of longitudinal data. The algorithm is a combination of the previously mentioned methods. Each longitudinal measured intermediate risk factor is summarized into a set of random slopes and random intercepts. The SNP variables are transformed via optimal scaling within each step of the algorithm and hereafter penalized, such that only a small part of the set of SNP variables is selected.

**Figure 4:**
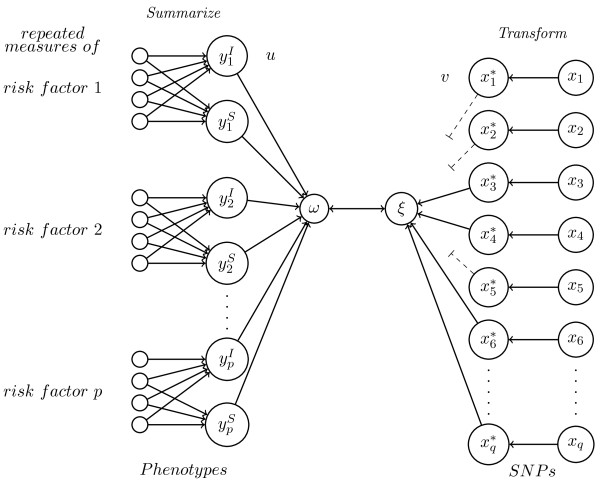
**Penalized nonlinear canonical correlation analysis for longitudinal data**. Each of the *p *longitudinal measured risk factors is summarized into a slope (*S*) and an intercept (*I*) variable. The SNP variables are transformed via optimal scaling within each step of the algorithm and hereafter penalized; SNPs that contribute little, based upon their weights (v) are eliminated (dotted lines) and the relevant variables remain. The obtained canonical variates *ω *and *ξ *correlate maximally.

Suppose we have two matrices, the *n *× *q *matrix **X**, containing the *q *SNP variables, and the *n *× *p *matrix **Y **containing the *p *summary measures of the intermediate risk factors. Then we want to optimize the weight vectors **u***' *= (*u*_1_,⋯,*u*_*p*_) and **v***' *= (*v*_1_,⋯,*v*_*q*_), such that the *n *× 1 canonical variate *ω *and the *n *× 1 canonical variate *ξ *correlate maximally. Then the algorithm is as follows (see figure 4):

1. Standardize **Y **(summarized intermediate risk factors).

2. Set k←0.

3. Assign arbitrary starting value to .

4. Estimate *ξ*, *ω*, **v **and **u **iteratively, as follows

Repeat

(a) k ← k+1.

(b)  (since X* is undefined for *k *= 1,  is as given in step 3).

(c) Compute  using weighted least squares

Set starting values **u **= (1,1,..,1)'.

**Repeat loop across variables ***j*, *j *= 1, ..., *p*

(a) Remove the contribution of all variables except variable *j*

(b) Obtain the estimate of 

(c) Update **u**, with 

**until u **has converged.

(d) Normalize  and set 

(e) Obtain the transformed matrix X* via optimal scaling. That is for each *j *(*j *= 1, ..., *q*)

with **G**_*j *_the *n *× *g*_*j *_indicator matrix for variable *j *with *g*_*j *_the number of categories of variable *j*. Restrict  to obtain . Then .

Standardize X*.

(f) Compute  using univariate soft-thresholding.

with *f*_+ _= *f *if *f *> 0 and *f*_+ _= 0 if *f *≤ 0.

(g) Normalize .

until  and  have converged

### Residual matrices

One canonical variate pair might not be enough to explain all the associations between the two sets of variables (**X **and **Y**), several other canonical variates can be obtained via the residual matrices; the part of the variables that is explained by the preceding pairs of canonical variates is removed from the sets. As long as either of the residual matrix of **X **or **Y **is determined the results remain the same [3]; it is easier to determine the residual matrix of **Y**, therefore, **Y**^*res *^= **Y **- *ωθ'*, where *θ *is the vector of linear regression weights of all Y-variables on *ω*. Optionally, to make sure each SNP variable can only be transformed in one optimal way, **X**^*res *^equals X* with the previously transformed variables fixed at their first optimal transformation.

### Cross-validation and permutation

Beforehand, the data is divided into two sets, one set functions as a test set to evaluate the performance of the final model, and the other (estimation) set is used to estimate the model parameters and to optimize the penalty parameter. Optimization of the penalty parameter for each canonical variate pair is determined by *k*-fold cross-validation. The estimation set is divided into *k *subsets (based upon subjects), of which *k *- 1 subsets form the training set and the remaining subset forms the validation set. The weight vectors **u **and **v **and the transformation functions ℑ_*j *_are estimated in the training set and are used to obtain the canonical variates in the training and validation sets. This is repeated *k *times, such that each subset has functioned both as a validation set and part of the training set.

Instead of determining the penalty, it is for sake of interpretation easier to determine the number of variables to be included in the final model [2,17,18]. We determined within each iteration step, the penalty that corresponded with the selection of the predetermined number of variables and penalized accordingly. The optimization criterion minimized the absolute mean difference between the canonical correlation of the training and validation sets [3];

Here  and  are the weight vectors estimated by the training sets,  and **Y**_-*j *_in which subset *j *was deleted and  the transformed validation set following the transformation of the training set . By varying the number of variables within the set of SNPs, the optimal number of variables which minimizes the optimization criterion is determined.

If the number of variables is large, there is a high probability that a random pair of variables has a high correlation by chance, while there is no correlation in the population. Because the canonical correlation is at least as large as the largest observed correlation between a pair of variables, the canonical correlation can be high by chance as well. To identify a canonical correlation that is large by chance only, we performed a permutation-analysis on the validation sets. We permuted the canonical variate *ξ *(SNP-profile) and kept the canonical variate *ω *(summary measures) fixed and then determined the difference between the canonical correlation of the training and the permuted validation sets; this was compared with the difference between the canonical correlation of the training and of the non-permuted validation sets. The closer they are together, the higher the chance that the model does not fit well.

## Competing interests

The authors declare that they have no competing interests.

## Authors' contributions

SW developed the algorithms, carried out the statistical analyses, and drafted the manuscript, AHZ carried out the statistical analyses and drafted the manuscript. Both authors read and approved the final manuscript.
